# Perinatal bisphenol A exposure promotes dose-dependent alterations of the mouse methylome

**DOI:** 10.1186/1471-2164-15-30

**Published:** 2014-01-17

**Authors:** Jung H Kim, Maureen A Sartor, Laura S Rozek, Christopher Faulk, Olivia S Anderson, Tamara R Jones, Muna S Nahar, Dana C Dolinoy

**Affiliations:** 1 Department of Environmental Health Sciences, University of Michigan, 1415 Washington Heights, Ann Arbor, Michigan, USA; 2 Department of Computational Medicine and Bioinformatics, Medical School, University of Michigan, Ann Arbor, Michigan, USA; 3 Department of Otolaryngology, Medical School, University of Michigan, Ann Arbor, Michigan, USA

**Keywords:** Bisphenol A, DNA methylation, Environmental epigenomics, MethylPlex

## Abstract

**Background:**

Environmental factors during perinatal development may influence developmental plasticity and disease susceptibility via alterations to the epigenome. Developmental exposure to the endocrine active compound, bisphenol A (BPA), has previously been associated with altered methylation at candidate gene loci. Here, we undertake the first genome-wide characterization of DNA methylation profiles in the liver of murine offspring exposed perinatally to multiple doses of BPA through the maternal diet.

**Results:**

Using a tiered focusing approach, our strategy proceeds from unbiased broad DNA methylation analysis using methylation-based next generation sequencing technology to in-depth quantitative site-specific CpG methylation determination using the Sequenom EpiTYPER MassARRAY platform to profile liver DNA methylation patterns in offspring maternally exposed to BPA during gestation and lactation to doses ranging from 0 BPA/kg (Ctr), 50 μg BPA/kg (UG), or 50 mg BPA/kg (MG) diet (N = 4 per group). Genome-wide analyses indicate non-monotonic effects of DNA methylation patterns following perinatal exposure to BPA, corroborating previous studies using multiple doses of BPA with non-monotonic outcomes. We observed enrichment of regions of altered methylation (RAMs) within CpG island (CGI) shores, but little evidence of RAM enrichment in CGIs. An analysis of promoter regions identified several hundred novel BPA-associated methylation events, and methylation alterations in the *Myh7b* and *Slc22a12* gene promoters were validated. Using the Comparative Toxicogenomics Database, a number of candidate genes that have previously been associated with BPA-related gene expression changes were identified, and gene set enrichment testing identified epigenetically dysregulated pathways involved in metabolism and stimulus response.

**Conclusions:**

In this study, non-monotonic dose dependent alterations in DNA methylation among BPA-exposed mouse liver samples and their relevant pathways were identified and validated. The comprehensive methylome map presented here provides candidate loci underlying the role of early BPA exposure and later in life health and disease status.

## Background

**Figure 1:**
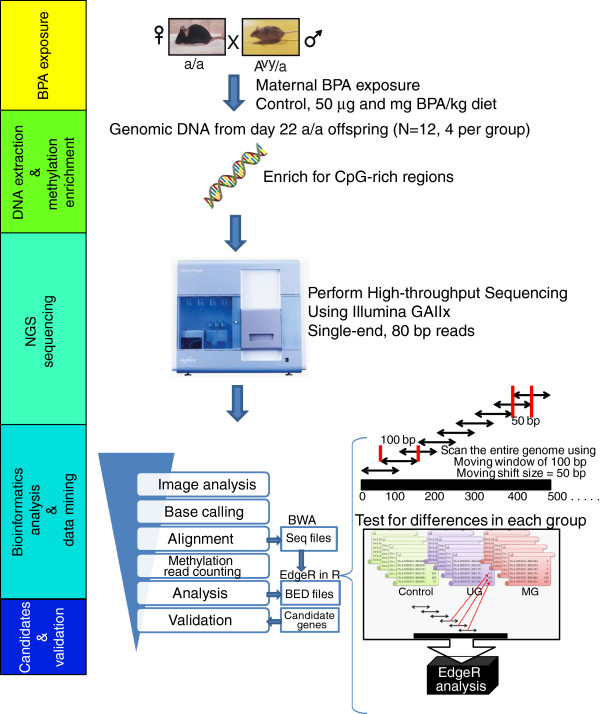
**Experimental and bioinformatics pipeline.** Next generation sequencing was performed after methylation-sensitive enzyme-based enrichment using the Illumina Genome Analyzer IIx (GAIIx) platform with single-end read length of 80 bp on 12 genomic liver DNA samples obtained from isogenic day 22 *a/a* mice, exposed to BPA through maternal dietary intake (control, 50 ug/kg diet, or 50 mg/kg diet; n = 4 per group). To regions of altered methylation (RAMs) associated with BPA exposure group, the number of aligned sequencing reads per 100 bp window with 50 bp moving shifts was obtained for each sample. edgeR analysis was performed after applying filters to remove the regions with low reads. Top candidates were identified, and candidate RAMs were validated using EpiTYPER.

The early developmental environment is an influential predictor of subsequent phenotypes and disease risk later in life. A growing body of work supports the “developmental origins of health and disease” (DOHaD) hypothesis, which posits that chemical and/or nutritional influences during early life result in long-lasting effects and point to epigenetic inheritance as a prime mechanism [1,2]. Epigenetic modifications, such as DNA methylation and chromatin markings, are established early in development and can shape susceptibility to disease, resulting in diverse phenotypes among genetically identical individuals [3]. Until recently, however, most attempts to elucidate the effects on the epigenome following environmental and nutritional exposures were either candidate gene driven or based on epigenetic techniques with limited genome coverage/sensitivity. Using bisphenol A (BPA) as a representative early environmental exposure alongside an established dose-dependent mouse model of perinatal exposures [4-7], we have developed a comprehensive strategy for evaluating environmental effects on the developing epigenome (Figure 1).

BPA is a high-production volume monomer used in the manufacture of polycarbonate plastic and epoxy resins. It is present in products that are routinely used, including food and beverage containers, baby bottles, dental composites, and thermal receipt paper [8]. Several studies have reported detectable levels of total urinary BPA in a large proportion of populations around the world [9-11]. An evaluation of circulating blood BPA levels in pregnant women in southeast Michigan indicated exposure levels between 0.5 μg/L and 22.3 μg/L (mean 5.9 μg/L) [12], and our recent study of human fetal liver samples indicated that there is considerable exposure to BPA during pregnancy and that BPA in fetuses was in a unconjugated form not readily eliminated from the body [13]. These findings indicate that *in utero* development and infancy may be particularly vulnerable time periods for exposure to BPA.

Toxicology studies indicate BPA exposure, both at high levels and levels well below the established U.S. Environmental Protection Agency (EPA) reference dose of 50 μg/kg body weight/day, results in a variety of physiological changes implicated in breast and prostate cancer, reproductive dysregulation, and behavioral abnormalities [14,15]. Epidemiology studies have described associations between increased BPA levels with cardiovascular disease risk, decreased semen quality, altered childhood behavior, and recurrent miscarriages [16-19]. BPA can mimic or antagonize endogenous hormones by binding weakly to steroid receptors including estrogen receptors (ER α and β) and thyroid hormone receptor [20-22]. BPA also binds strongly to the trans-membrane ER, G protein-coupled receptor 30 (GPR30), as well as the orphan nuclear receptor estrogen related receptor gamma (ERRγ) [23,24], and can also activate transcription factors, including peroxisome x receptor (PXR) and aryl hydrocarbon receptor (AhR), which can dimerize with steroid receptors [25,26].

BPA is associated with epigenetic alterations following developmental exposures [4,5,27-30]. In a rat model, Ho and colleagues observed multiple changes in gene-specific DNA methylation patterns in the adult male prostate, including hypomethylation of the phosphodiesterase type 4 variant 4 (*Pde4d4*) [27]. Hypomethylation of the nucleosome binding protein-1 (*Nsbp1*) gene promoters and hypermethylation of the hippocalcin-like 1 (*Hpcal1*) gene promoter was also reported in rats following neonatal exposure to low concentrations of BPA (10 μg/kg of body weight BPA) [30]. Altered methylation and subsequent aberrant gene expression was associated with a marked increase in prostate cancer risk. Using the viable yellow agouti (A^vy^) mouse model, we have shown that maternal dietary exposure to moderate levels of BPA (50 mg BPA/kg diet) resulted in decreased DNA methylation at the *A*^*vy*^*,* and *Cabp*^*IAP*^ metastable epialleles [4,5], while exposure to lower doses (50 ng and 50 μg BPA/kg diet) led to hypermethylating effects at these candidate loci [5]. Finally, using restriction-enzyme based methylation technology, Yaoi and colleagues reported both hyper- and hypomethylation at a methylation-sensitive NotI loci in murine offspring forebrain following gestational exposure to 20 μg/kg body weight of BPA [28]. Recently, the differential methylation in imprinting control regions was reported in maternally BPA-exposed mouse embryos and placentas using pyrosequencing technology. This change in methylation also resulted in abnormal expression in placenta and abnormal placental development [31].

Capitalizing on advances in whole-genome epigenomic and high-throughput quantitative DNA methylation technologies, we developed a comprehensive approach to identify the constellation of genomic loci with altered epigenetic status following dose-dependent perinatal BPA exposure. Using a tiered focusing approach, our strategy proceeded from unbiased broad DNA methylation analysis using methylation-based next generation sequencing technology to in-depth quantitative site-specific CpG methylation determination using the Sequenom EpiTYPER MassARRAY platform. We compared the regions of altered methylation (RAMs) following BPA exposure using bioinformatics and biostatistics methods, and the cellular pathways in which the genes with nearby RAMs function.

## Results

### Analysis pipeline and quality control for identifying differential methylation

We used the MethylPlex-Next Generation Sequencing (M-NGS) platform to evaluate genome-wide alterations in DNA methylation following perinatal BPA exposure in mice, which requires minimal DNA input (~ 50 nanograms) and enriches methylated DNA using a cocktail of methylation-dependent restriction enzymes prior to deep sequencing (Figure 1). Following alignment to the reference mouse genome (version mm9), we confirmed that MethylPlex library reads were enriched in genomic regions containing higher numbers of genes and CpG islands (CGIs) (Additional file 1: Figure S1). For initial standardization of the data analysis pipeline, we employed a sex-based analysis comparing methylation profiles on chromosomes X and Y between female and male offspring (Additional file 1: Figure S2). The difference in mapped reads on chromosomes X and Y was clearly distinguishable between male and female samples with minimal background noise observed on chromosome Y from female samples. Upon examination of the chromosomal distribution of windows with significant differential methylation applying the same criteria employed in the exposure comparison strategy outlined in the Methods, 263 and 325 windows (total = 696) were located on chromosome X and Y, respectively, compared with only 108 windows on autosomes (Additional file 1: Figure S3). Despite the presence of a limited number of background reads on chromosome Y in female samples, no regions on this chromosome were identified to harbor hypermethylation in female samples. This analysis provides us an estimate of the maximum false discovery rate (FDR) of 15.5% (108/696 * 100%) for our analysis presented below; however, the actual FDR may be much lower, if true autosomal differences in methylation exist between sexes.

### BPA Exposure Dependent Regions of Altered Methylation (RAMs)

When genome-wide DNA methylation patterns were compared across BPA exposure categories (control vs. MG, control vs. UG, and UG vs. MG), a small percentage (0.2 to 0.3%) of windows (106,765, 166,437, and 173,823 windows, respectively, in over 53 million windows of 100 bp with 50 bp moving-shifts) were identified as preliminary regions of altered methylation (RAMs) (p-value & 0.05; Additional file 1: Figure S4A) prior to applying additional filtering steps described in Methods. Across the three BPA exposure comparisons, a majority of RAMs (82%, which is 310,024 out of 378,371 in 100 bp windows) were distinct from one another (Additional file 1: Figure S4B). RAMs were identified both within and outside of CGIs, CGI shores (0-2 kb from CGI), and CGI shelves (2-4 kb from CGI) (Additional file 1: Figure S4C).

**Figure 2:**
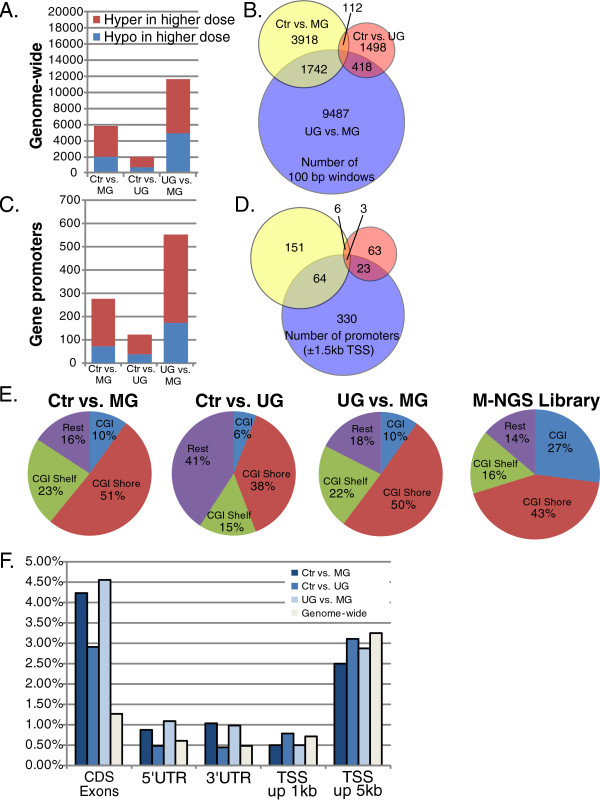
**Characterization of genome-wide methylation in BPA-exposed offspring.** The genomic distributions of regions of altered methylation (RAMs), delineated as genome-wide or within promoter regions, from the edgeR analysis with p-value & 0.05 are displayed. **(A)** The bar graph identifies the number of RAMs in genome-wide scale. **(B)** The Venn diagram displays the overlap of RAMs among control (Ctr), 50 μg (UG) and 50 mg (MG) diet groups. **(C)** The bar graph and **(D)** Venn diagram of RAMs within promoter regions is displayed. **(E)** For each exposure comparison and for the full M-NGS library, pie charts display the proportion of RAMs in number of windows at CpG islands (CGIs), CpG shores, CpG shelves, and the remainder of the genome. In the Ctr vs. MG and in the UG vs. MG comparisons, approximately half of the RAMs occur in CpG shores (0-2 kb from CGIs). **(F)** The genomic distributions of RAMs (p-value & 0.05) at 5′UTRs, TSSs, CDSs, and 3′UTRs are delineated in bar graphs. The percentage of RAMs at CDS exons in the Ctr vs. UG comparison is decreased by more than half compared to other group comparisons. At the TSSs, the percentage of RAMs is comparable among the three group comparisons.

To minimize the influence of a single sample in predicting RAMs, we further analyzed data with filtered RAMs that 1) exhibited methylation change in at least two samples per exposure category and 2) displayed differential methylation either in at least one out of two flanking windows (shift window size is 50 bp) or two 100 bp windows within a 500 bp stretch. These filtering steps were used for the male versus female comparison and thus are expected to result in an FDR no greater than 15.5%. We then conducted a refined downstream analysis, similar to the unfiltered analysis described above. Following filtering, within each exposure comparison we observed a greater number of hypermethylated RAMs compared to hypomethylated RAMs (Figure 2A and C). The largest number of RAMs was observed when UG exposed offspring were compared to MG exposed offspring (N = 11,647 genome-wide, and N = 428 promoter-region only, Figure 2A and C). The control versus UG exposure category resulted in the smallest number of RAMs (N = 2,028 genome-wide, and N = 93 promoter-region only), while the control versus MG exposure category resulted in 5772 genome-wide and 227 promoter region RAMs (Figure 2A and C). Similarly to the unfiltered analysis above, across the three BPA exposure categories, RAMs were largely distinct from one another (Figure 2B and D).

Using the filtered dataset, we also examined the distribution of RAMs among CGIs, CGI shores (0-2 kb from CGI), and CGI shelves (2-4 kb from CGI), and compared these to the proportion of the M-NGS library and mouse \genome (mm9) covered by CGIs, CGI shores, and CGI shelves. The distribution of CGIs, CGI shores, and shelves in the mouse genome (mm9) is shown in Additional file 1: Figure S5, where less than half of the genome was shown to be associated with CGIs, CGI shores, or shelves. In the M-NGS libraries enriched for CGs, over 85% of the reads were associated with CGIs, CGI shores, or shelves (43% in CGI shores, 27% in CGIs, and 16% in CGI shelves). Less than 15% of the reads were located outside of CGIs and their surrounding area (14%) (Figure 2E). Approximately half of the total differential regions were located within CGI shores in the Ctr vs. MG (51%) and the UG vs. MG (50%) comparisons, followed by CGI shelves, which accounted for over 20% of the total differential regions (Figure 2E). In the Ctr vs. UG comparison, however, a smaller proportion of the differential regions were located within CGI shores (38%) and shelves (15%) (Figure 2E). The relative distribution of CGIs, shores, and shelves of the RAMs in comparison with the M-NGS library identified a slight enrichment of RAMs in CGI shores (18.6% increase in Ctr vs. MG and 16.3% increase in UG vs. MG) and CGI shelves (43.7% increase in Ctr vs. MG and 37.5% increase in UG vs. MG), and depletion of RAMs (63% decrease in Ctr vs. MG and UG vs. MG, and 78% decrease in Ctr vs. UG) in CGIs. In the Ctr vs. UG comparison, the relative distribution was decreased in CGI shores by 11.6%, in contrast with Ctr vs. MG and UG vs. MG comparisons with an increase in relative distribution of CGI shores. These results identified the CGI shores and shelves to be the more susceptible and CGIs to be more resistant to methylation changes upon environmental exposure. Additional pie charts in Additional file 1: Figure S5 display the proportion of hyper- and hypo-methylated regions with respect to CGIs, CGI shores, and shelves.

In addition, we examined the distribution of epigenetic changes within various genomic locations including exons, 5′ and 3′ untranslated regions (UTRs), and within 1 and 5 kb of transcription start sites (TSSs) upon various BPA exposures using RSeQC package [32]. In Ctr vs. MG and UG vs. MG analyses, the genomic distribution of differentially methylated regions showed more than 3-fold enrichment of coding sequence (CDS) exons (3.3 fold increase in Ctr vs. MG and 3.6-fold increase in UG vs. MG) compared to background levels in the mouse genome (Figure 2F). In addition, the enrichment of 5′ (1.4-fold increase in Ctr vs. MG and 1.8-fold increase in UG vs. MG) and 3′UTRs (2.1-fold increase in Ctr vs. MG and 2-fold increase in UG vs. MG) and the depletion of the upstream TSSs (1.4-fold decrease in both Ctr vs. MG and UG vs. MG for 1 kb and 1.3-fold decrease in Ctr vs. MG for 5 kb) was observed. In the Ctr vs. UG analysis, however, the genomic distribution difference between the RAMs and the mouse genome background was not observed, except for a 2-fold increase in CDS exons. Despite the small overlap of RAMs between Ctr vs. MG and UG vs. MG comparisons, the genomic and CGI distributions of the differential regions were highly similar, and unlike the Ctr vs. UG comparison, the results deviated from the genomic background.

### Promoter regions associated with BPA-dependent regions of altered methylation

**Figure 3:**
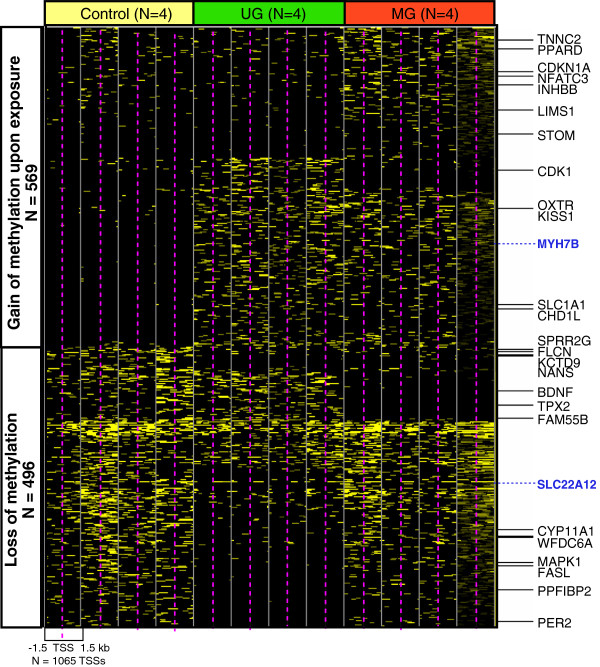
**Regions of Altered Methylation (RAMs) in gene promoters.** RAMs within ±1.5 kb flanking transcription start sites (TSSs) are represented. Each column separated by gray lines represents a single sample (4 per group), and each row represents a unique gene promoter region, ±1.5 kb from the TSSs (magenta dotted line). The known BPA-interacting genes queried from the Comparative Toxicogenomic Database are also indicated on the right. Two arrows pointing to the *Myh7b* and *Slc22a12* gene promoters (in blue text) show candidate RAMs validated in a larger subset of animals.

Differential methylation in promoter regions may play a substantial role in gene transcriptional regulation. The identified RAMs in promoter regions (N = 1,065, p-value & 0.05) that occur within ±1.5 kb from TSSs are visualized in Figure 3. Fifty-three percent of RAMs gained methylation at promoters (N = 569), and forty-seven percent lost methylation (N = 496) upon BPA exposure. Promoter RAMs can be further classified into types that respond to UG exposure, respond to MG exposure, or respond to both exposures. For RAMs with a gain of methylation, only a small proportion of the TSSs were associated with the UG exposure only (N = 60). Thus, gains of methylation upon exposure were either observed in both the UG and MG exposure groups (N = 277) or in only the MG exposure group (N = 232). For RAMs with a loss of methylation, a large proportion of the TSSs were affected by the UG exposure only (N = 363), while only a small number of TSSs showed differential methylation upon both UG and MG exposure groups (N = 44) or only in the MG exposure group (N = 89).

### Enriched gene ontology terms and pathways among BPA exposure dependent differentially methylated genes

**Figure 4:**
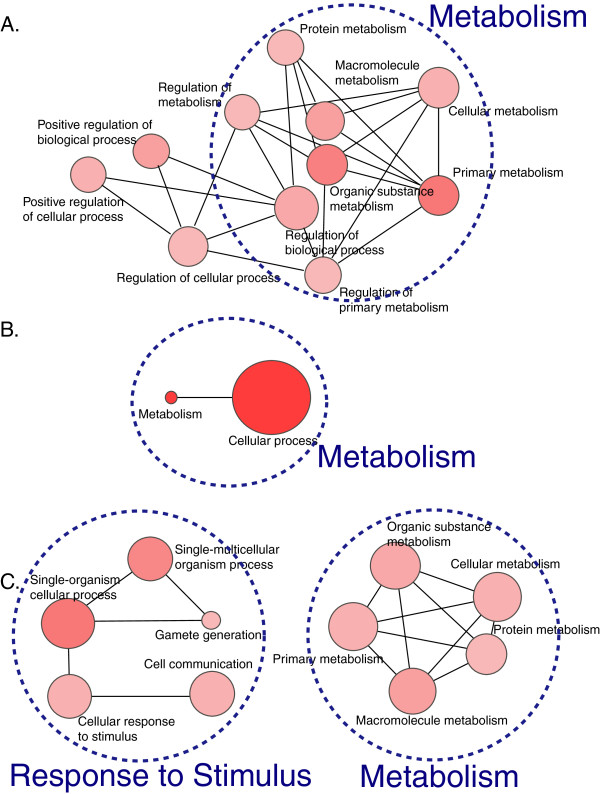
**Enriched biological processes among genes harboring differential methylation.** The genes harboring differential methylation within their promoters (FDR adjusted p-value & 0.05) were subjected to GO term enrichment analysis. **(A)** A total of 198 genes from Ctr vs. MG comparison, **(B)** 76 genes from Ctr vs. UG comparison, and **(C)** 371 genes from UG vs. MG comparison are used for the analysis, and enriched pathways are involved in basic biological processes, including basic cellular, metabolic, and immune and stimulus responses, as well as some binding activities. The enriched biological processes are graphed using REViGO software. The colors of the circles represent the various levels of statistical significance, where the darker shade represents more significance in p-values than the lighter one. The various sizes of the circles represent the number of genes in given GO terms. A complete list of GO terms including cellular components and molecular functions is available in Additional file 2: Table S4.

We examined the enrichment of Gene Ontology (GO) terms and pathways present in our candidate regions within 1.5 kb of a TSS (N = 19,720; Additional file 2:Table S3) using the Gene Set Enricher application from the Comprehensive Toxicogenomics Database (CTD) site, and the results were visualized using the REViGO web application (Figure 4). GO biological processes enriched for BPA-exposure RAMs in Ctr vs. MG comparison (n = 198 genes, FDR & 0.05) included metabolism and stimulus response (Figure 4A; Additional file 2: Table S4). Only 4 significant GO molecular functions were observed, and they were involved in general binding activities (binding, protein and ion binding, catalytic activity, FDR & 0.05). The significant pathways altered include *transmembrane transport of small molecules* (REACT:15518) and *metabolism* (REACT:111217). In the Ctr vs. UG comparison, 76 genes were assessed, and two GO biological process terms include metabolic process and cellular process (Figure 4B). In addition, *cancer related pathways* were enriched (KEGG:05200) (Additional file 2: Table S4). For the UG vs MG comparison, a total of 371 genes were assessed, and we observed strong enrichment of GO terms involved in metabolic processes and stimulus as well as signaling processes (Figure 4C). The significant pathways include *glutamatergic synapse* (KEGG:04724) and *regulation of autophagy* (KEGG:04140) (Additional file 2: Table S4).

Enriched GO terms and pathway analysis was also performed on 156 known BPA-interacting genes (curated from the CTD), that are known to be expressed in the mouse liver from Mouse Genome Informatics Gene Expression Database and compared with the results from our methylation data (Additional file 2: Table S5). This analysis identified 67 pathways and 912 GO terms that are significantly enriched among BPA-interacting genes, representing genes whose altered DNA methylation may be associated with concomitant gene expression changes in the liver. Some of the top pathways include *pathways in cancer* (KEGG:05200, FDR & 2.77e-28), *metabolism* (REACT:111217, FDR & 2e-21), and *adipocytokine signaling pathway* (KEGG:04920, FDR & 1.56e-10). By identifying enriched pathways using two lists, including one focusing on epigenetically altered genes and another on transcriptionally regulated genes, the similarities as well as the differences between the affected pathways via two different mechanisms can be compared.

In addition to our promoter based enrichment analysis above, whose differential sites were restricted to within 1.5 kb of TSSs, we also performed pathway enrichment analysis with all RAMs using ChIP-Enrich (http://chip-enrich.med.umich.edu). The ChIP-Enrich application assigns peaks to genes based on a chosen method (we used nearest TSS) and tests peaks from ChIP-seq experiments for enrichment of biological pathways, GO terms, and other types of gene sets using an empirical method to adjust for the relationship between probability of a peak and the genomic length associated with a gene. Associating genomic sites or peaks to nearest TSSs has been widely applied in the biological functional analysis of ChIP-Seq data [33]. In the Ctr vs. MG and in the UG vs. MG comparisons, similar pathway enrichments were obtained as seen in our promoter region based testing, mainly metabolism and its related processes (FDR & 0.01), as well as GO terms related to development and morphogenesis (FDR & 0.01) (Additional file 2: Table S6). In the Ctr vs. UG enrichment results, only 7 GO terms were significant with FDR & 0.1, while there are 109 terms enriched in Ctr vs. MG, and 119 terms enriched in UG vs. MG comparisons (FDR & 0.1). Three of the top 7 enriched GO terms were lipoprotein particle receptor activity (FDR & 0.04), low-density lipoprotein receptor activity (FDR & 0.07), and apolipoprotein binding (FDR & 0.09).

### Validation of regions of altered methylation using sequenom EpiTYPER

**Figure 5:**
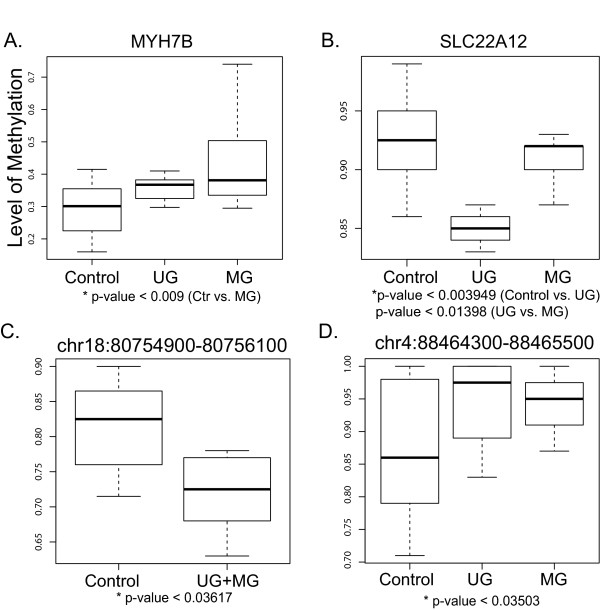
**Candidate region validation using Sequenom EpiTYPER.** RAMs from candidate regions including **(A)***Myh7b* and **(B)***Slc22a12* promoters and **(C, D)** two intergenic regions from 29 mouse liver samples including the 12 original samples sequenced for M-NGS were quantitatively validated via Sequenom EpiTYPER and shown as box plots.

RAMs from 5 genomic regions were quantitatively validated using the Sequenom EpiTYPER platform. We validated two RAMs located in the gene promoters of myosin, heavy chain 7B, cardiac muscle, beta (*Myh7b*) (p-values & 0.006, 300 bp windows) and renal-specific transporter (*Slc22a12*) (p-values & 0.009, 300 bp windows) (Additional file 2: Table S7). Both of these genes were associated with metabolic process in our ChIP-Enrich testing (FDR & 1.6E-4 and 2.9E-5 in UG vs. MG and Ctr vs. MG comparisons). Several enriched concepts involved in binding processes such as ribonucleotide, nucleotide, actin, and cytoskeletal protein bindings (FDR & 0.05) in our ChIP-Enrich analysis were associated with Myh7b, and those involved in transport activities and nitrogen metabolic process (FDR & 0.05) were associated with Slc22a12. The methylation gain in the promoter region of *Myh7b* in both the UG and MG exposures (  3) was validated, showing a median methylation of 30.1% (22.8 for Quartile 1, Q1 and 35.4 for Quartile 3, Q3) in Ctr compared to 36.8% (32.5 for Q1 and 38.3 for Q3) in UG and 38.1% (34.3 for Q1 and 48.6 for Q3) in MG (Figure 5A). The gene expression change in *Myh7b* was monitored using real-time qPCR, revealing no change in expression in PND22 mouse livers. The hypomethylation in the *Slc22a12* promoter region in the UG exposure group (Figure 3) was confirmed with a median methylation level across four CpG sites observed at 90% (89.5 for Q1 and 92 for Q3) in Ctr, 84% (82.1 for Q1 and 88.8 for Q3) in UG, and 89% (85.5 for Q1 and 92.9 for Q3) in MG. The decrease in methylation at CpG site 2 in UG exposure group was statistically significant compared to Ctr (p-value & 0.004) and MG (p-value & 0.01) groups (Figure 5B). The gene expression change in *Slc22a12* was assayed via qPCR, revealing that this gene is not expressed in PND22 mouse liver tissue.

Additionally, we measured methylation in three RAMs in intergenic regions from chromosome 1, 4, and 18 to confirm differential methylation associated with BPA exposure. Hypomethylation was validated in the region from chromosome 18 (p-value & 0.036) (Figure 5C), with a median methylation level of 82.5% (77.1 for Q1 and 86 for Q3) in the Ctr group compared to 72.5% (68 for Q1 and 77 for Q3) in UG and MG BPA group. The intergenic region from chromosome 4 was hypermethylated as indicated by the M-NGS (p-value & 0.035) with a median methylation of 86% (79 for Q1 and 98 for Q3) in the Ctr group, compared to 97.5% (92 for Q1 and 100 for Q3) in the UG BPA group, and 95.0% (91 for Q1 and 97.5 for Q3) in the MG group (Figure 5D). The remaining region on chromosome 1 that showed a M-NGS identified loss of methylation with BPA exposure was not differentially methylated following bisulfite sequencing validation (p-value > 0.35), with a median methylation of 85.7% (77.7 for Q1 and 90.8 for Q3) in Ctr, 82.7% (81.7 for Q1 and 85.3 for Q3) in UG, and 87% (84.7 for Q1 and 91 for Q3) in MG groups.

## Discussion

We have previously shown that BPA exposure at 50 mg BPA/kg diet during development plays a role in epigenetic programming at candidate metastable loci *A*^*vy*^ and *Cabp*^*IAP*^[4]. In a follow-up study using multiple doses of dietary BPA exposures, we observed dose-dependent effects on DNA methylation at *A*^*vy*^ and *Cabp*^*IAP*^ with the lower doses (50 μg and 50 ng BPA/kg diet) leading to the opposite, hypermethylating effect [5]. We have now employed a next-generation sequencing approach and identified non-monotonic effects on the DNA methylome following human physiologically relevant perinatal BPA exposures. The identification of low dose and non-monotonic effects of endocrine disrupting chemicals, such as BPA, is a topic of growing interest in toxicology and endocrinology [15].

Genome-wide platforms allow for identification of the constellation of genomic loci with altered epigenetic status following exposure or in relation to disease status. For example, Irizarry et al. used a microarray approach and demonstrated that approximately 70% of methylation changes in colon cancer samples occurred in “shores” defined as regions up to 2 kb away from CpG islands [34]. Newer approaches to query the methylome now involve next-generation sequencing, and the technology in this field is rapidly advancing, including whole genome and reduced representation bisulfite sequencing, which quantitatively measure methylation changes at single-based resolution, yet remain costly in addition to the need for complex bioinformatics pipelines. An alternative to bisulfite conversion approaches include affinity enrichment techniques (e.g. MeDIP-seq and MBD-seq) that involve the enrichment of methylated regions via methylation-targeted antibody or protein. These methods, however, are sensitive to antibody lot that may lead to inconsistent enrichment between experiments. In this project, we employed the MethylPlex-Next Generation Sequencing (M-NGS) platform, which uses enzymatic enrichment to identify regions of altered methylation and requires only 50 ng of starting genomic material. Because the exact composition of the enzymes used for the methylation enrichment is proprietary information (Patent Number US 2007/0031858 A1), we assessed CG enrichment prior to downstream analysis and confirmed an average of 2.3 fold CG enrichment across our 12 study samples compared to mouse reference genomes using this technology. In addition, we have previously assessed and published on the performance of the MethylPlex platform on CG enrichment in prostate cancer cell lines and tissues [35].

Our genome-wide analysis of liver DNA from mouse offspring exposed to BPA indicates that DNA methylation patterns exhibit non-monotonic effects following perinatal exposure to BPA, corroborating previous studies using multiple doses of BPA with non-monotonic outcomes [36,37]. We observed an enrichment of RAMs in CGI shores, accounting for nearly half of the identified RAMs in MG BPA group compared to either control or UG BPA groups. This suggests that CGI shores and regions outside of often-profiled CpG islands may be more susceptible to epigenetic changes following perinatal exposures. For example, RAMs identified in the higher BPA exposure group (MG) were more likely to be located within CGI shores, and CGIs were more resistant to epigenetic change. In the lower BPA exposure group (UG), however, an enrichment of the RAMs among CGI shores was not observed; instead regions with low CG density were highly enriched as RAMs. In addition, the overall distribution of the RAMs within CDS, UTRs, and TSSs was also distinct between MG and UG exposed samples.

In order to identify gene promoters with altered DNA methylation upon exposure, we scanned ±1,500 bps flanking the TSSs of 30,637 transcripts in the mouse genome (mm9). This analysis indicated distinct exposure-dependent methylation patterns around TSSs (span of 3,000 bp) and identified several hundred novel BPA-induced promoter methylation events. Several of the identified promoter methylation events occurred in genes previously associated with transcriptional change following BPA exposure, including *Hmgn5, Hpcal1, Hoxa10, Brca1, Pde4d*[27], and *Esr1* and *Esr2*[29]. In addition, decreased promoter methylation and increased expression were reported in high mobility group nucleosome binding domain 5 (*Hmgn5*) from the prostate of male adult rat neonatally exposed to 10 μg BPA/kg diet [30], and in Homeobox protein Hox-A10 (*Hoxa10*) from the reproductive tract of CD-1 mice neonatally exposed to 5 mg BPA/kg diet [38]. Increased promoter methylation and decreased expression in Hippocalcin-like protein 1 (*Hpcal1*) in new born male rats exposed to 10 μg BPA/kg diet [30] and breast cancer type 1 susceptibility protein (BRCA1) in human mammary epithelial cells exposed to BPA for 1 week at the early passage [39] have also been reported. Using the Comparative Toxicogenomics Database (CTD), we identified 25 genes with previously reported changes in gene expression upon BPA exposure that also harbored aberrant DNA methylation near promoters in our BPA-exposed mouse liver samples (Figure 3).

To perform technical validation as well as to identify true differential methylation target genes upon BPA exposure, the original 12 samples along with 17 additional samples were included in the validation set. Two of the validation loci were located within gene promoter regions, and thus an alteration in methylation upon BPA exposure may result in concomitant gene expression changes. One of our candidate genes that gained methylation upon BPA exposure in our M-NGS data was *Myh7b*. Quantitative and CpG site specific validation using the Sequenom EpiTYPER platform confirmed the increase in DNA methylation within the promoter region of *Myh7b* in a monotonic dose-dependent manner (e.g. the higher the BPA exposure, the higher the methylation level). The MYH7B protein is known to interact with ESR2 [40], and one of the MYH7B estrogen-response elements (ERE) (http://www.genomatix.de) is located within an identified RAM. Despite the validated quantitative change in methylation in the *Myh7b* promoter, no exposure dependent alteration in expression was observed in PND22 mouse liver samples. During development, genes exhibit unique time windows of expression, and it’s possible a change in expression may have been missed or could occur at a future time point. Alternatively, the observed altered methylation upon BPA exposure may merely be an effect on the epigenome that will not manifest itself in a change in expression, protein level, or protein activity. *Slc22a12* is a candidate RAM displaying decreased level of methylation upon BPA exposure. In humans, the presence of single nucleotide polymorphisms (SNPs) in the *SLC22A12* gene was found to be associated with obesity and metabolic syndrome in Caucasians with hypertension [41]. As in the M-NGS data, a significant decrease in DNA methylation was observed in samples in the UG exposure group, but not in the MG exposure group, adding to the weight of evidence supporting non-monotonic epigenetic responses following BPA exposure.

Our pathway analysis indicated strong enrichment of genes involved in metabolism and stimulus response upon BPA exposure. This observation, in combination with previously reported data supporting a role for BPA in immune [42-45] and metabolic response [6,46-49], indicates the importance of changes in epigenetic pathways following perinatal exposures as a mechanism linking developmental exposures to disease risk in adulthood. For example, the activity of the adiponectin gene, which codes for a hormone controlling insulin sensitivity, was previously shown to be suppressed by BPA [49], implicating BPA in the development of type 2 diabetes. Stimulus response upon BPA exposure was previously identified in a prenatally BPA-exposed mouse with increased regulation of T helper 1 and 2 immune responses [43]. Estrogen is a known regulator of the immune response through various activities including the secretion of interferon-Ɣ and cytokine [50,51]. Several immune response experiments of environmental exposures including BPA have been previously conducted; in a mouse study, female offspring of mothers exposed to > 50 μg BPA/kg had elevated lung inflammation, compared with offspring of control dams [42]. Further, prenatal exposure to 10 μg BPA/mL in drinking water enhanced allergic sensitization and bronchial inflammation and responsiveness in a susceptible animal model of asthma [45].

To understand the full extent of BPA and associated perinatal exposures on the epigenome as a whole, it will be important to incorporate genome-wide analysis other epigenetic mechanisms such as histone modifications and non-coding RNAs, as well as full transcriptome analyses, such as RNA-seq. Indeed, we have recently identified DNA methylation and histone modifications to act in concert with one another at the *A*^*vy*^ metastable epiallele [52]. Increasing the number of studies focusing on multiple epigenetic mechanisms will strengthen the understanding of environmentally induced alterations to the epigenome.

## Conclusions

It is increasingly recognized that environmental exposure to chemical, nutritional, and behavioral factors alters gene expression and affects health and disease by not only mutating promoter and coding regions of genes, but also by modifying the epigenome. The investigation of early environmental effects can inform the fields of toxicology and environmental epidemiology by elucidating the mechanisms underlying developmental exposure and disease risk later in life. The identification of epigenomic loci dysregulated in a dose-dependent manner will ultimately strengthen human health risk assessment and shape diagnostic and therapeutic strategies for disease. The mouse is a tractable and popular model for human diseases; however animal models for toxicology studies may not be the best choice for modeling the potential impact on the human genome if the repertoire of epigenetically labile genes is markedly species dependent. Additional toxicologically relevant animal models, including rats and sheep should also be considered for this approach along with parallel approaches in human tissues. Ultimately, researchers must integrate the layers of epigenetic changes with the windows of susceptibility to understand and generate the best prescriptions for human health and disease. The comprehensive methylome map presented here will further our understanding on the methylation targets of BPA exposure. Since epigenetic profiles, unlike genetic mutations, are potentially reversible, approaches for prevention and treatment, such as nutritional supplementation and/or pharmaceutical therapies, may have significant impact on disease trajectory and, ultimately, human health.

## Methods

### Mouse liver tissue samples

Mice were obtained from a colony that has been maintained with sibling mating and forced heterozygosity for the viable yellow agouti (*A*^*vy*^) and non-agouti (*a*) alleles for over 220 generations, resulting in a genetically invariant background [53]. To avoid effects associated with parity, virgin wild-type *a/a* dams, 6 weeks of age, were randomly assigned to one of three phytoestrogen free AIN-93G diets (diet 95092 with 7% corn oil substituted for 7% soybean oil; Harlan Teklad, Madison, WI): 1) standard diet (n = 11 litters); 2) standard diet supplemented with 50 μg BPA/kg diet (n = 9 litters); or 3) standard diet supplemented with 50 mg BPA/kg diet (n = 13 litters). All diet ingredients were supplied by Harlan Teklad except BPA, which was supplied by NTP (National Toxicology Program, Durham NC). The MG dosage is an order of magnitude lower than the dietary administered maximum non-toxic threshold in rodents (200 mg/kg body weight/day) [54], but, it is important to note, as previously reported, that the BPA dosages capture human physiologically relevant exposure [5,55].

Wild-type *a/a* dams were provided with their respective diet two weeks prior to mating with 8-week-old *A*^*vy*^*/a* males and housed in polycarbonate-free cages with *ad libitum* access to diet and BPA-free water. The dams remained on the assigned diets throughout pregnancy and lactation, after which offspring were sacrificed at post-natal day 22 (PND22). This mating scheme produces approximately 50% *a/a* genotype and 50% *A*^*vy*^*/a* offspring. For this study, liver DNA from a subset of *a/a* wild-type animals was analyzed for full methylome characteristics: 1) standard diet (Ctr, n = 4 offspring; 2 male and 2 female); 2) 50 μg BPA/kg diet (UG, n = 4 offspring; 2 male and 2 female); 3) 50 mg BPA/kg diet (MG, n = 4 offspring; 1 male and 3 female). To validate epigenome-wide DNA methylation findings, liver DNA from additional PND 22 *a/a* animals was evaluated including: 1) standard diet (n = 14 offspring; 9 male and 5 female); 2) 50 μg BPA/kg diet (n = 5 offspring; 3 male and 2 female); 3) 50 mg BPA/kg diet (n = 10 offspring; 4 male and 6 female). From these mice, total genomic DNA was isolated from liver tissue using buffer ATL, proteinase K, and RNase A (Qiagen Inc., Valencia, CA), followed by phenol-chloroform extraction and ethanol precipitation. DNA quality and concentration was assessed using a ND1000 spectrophotometer (NanoDrop Technology, Wilmington, DL).

Animals used in this study were maintained in accordance with the *Guidelines for the Care and Use of Laboratory Animals* (Institute of Laboratory Animal Resources, 1996) and were treated humanely and with regard for alleviation of suffering. The study protocol was approved by the University of Michigan Committee on Use and Care of Animals.

### M-NGS library generation

MethylPlex library synthesis and GC-enrichment service was obtained through a commercial service at Rubicon Genomics Inc., Ann Arbor, MI (Patent Number US 2007/0031858 A1) [56]. The ability of MethylPlex combined with next-generation-sequencing (M-NGS) to identify regions of altered methylation was previously evaluated using prostate cancer cell lines and tissues, and the detail of the M-NGS methods is provided in Kim et al. [35]. Briefly, fifty nanograms of genomic DNA were digested with a proprietary cocktail of methylation-sensitive restriction enzymes and then amplified by PCR with universal primers to create a MethylPlex library that is enriched for methylated DNA. MethylPlex DNA was then subjected to additional enzymatic treatment to deplete all non-GC-rich DNA sequences, purified and amplified in a second round of PCR. After purification, amplification adaptors were removed by a restriction enzyme digest, and the purified products were directly incorporated into the Illumina genomic DNA sequencing sample preparation kit procedure (Illumina Inc., San Diego, CA) at the end repair step, skipping the nebulization process. An adenine base was then added to the purified end repaired products using Klenow exo (3′ to 5′ exo minus) enzyme. The reaction product was purified, ligated to Illumina adaptors with DNA ligase and resolved on a 2% agarose gel. Gel pieces were excised at 400 base pair positions, and the DNA was extracted using Qiagen gel extraction kit (Qiagen Inc., Valencia, CA).

### M-NGS sequencing and alignment

The purified MethylPlex library was analyzed by Bioanalyzer (Agilent Technologies, San Diego, CA) before subjecting it to flow cell generation, where 10 nM of library was used to prepare flowcells with approximately 30,000 clusters per lane, with the sequencing performed by the University of Michigan DNA Sequencing Core. The raw sequencing image data obtained by Illumina GAIIx using 80 cycles of single ends were analyzed by the Illumina analysis pipeline. Around 30 million reads per sample (ranging between 27 to 37 million reads) were obtained, where approximately 70% of these were mapped uniquely to the mouse mm9 reference genome using Burrows-Wheeler Aligner (BWA) tool (Additional file 2: Table S1).

### Tiered approach edgeR analysis

We adopted a tiered-based profiling pipeline to identify regions of altered methylation (RAMs) by examining the locus-specific genome-wide methylation patterns associated with BPA exposure levels (Figure 1). First, we scanned the entire genome using a window size of 100 bp with a 50 bp moving shift, which accounts for over 53 million windows for each sample. The genomic regions containing at least 10 reads in 25% of the samples (~ 4 million windows) were then subjected to edgeR analysis, which we used to test for differences in each exposure group [57]. This step removed the regions with low reads (no methylated CGs present in our sequencing library). The edgeR analysis using R software was run using the glmFit function, which uses a negative binomial generated linear model, and identified the regions with differential methylation in three different comparisons; the methylation levels from the control group (n = 4) against the 50 μg BPA/kg diet group (Ctr vs. UG, n = 4), control group against 50 mg BPA/kg diet group (Ctr vs. MG, n = 4), and 50 μg BPA/kg diet group against 50 mg BPA/kg diet group (UG vs. MG). For downstream analysis, identified RAMs were restricted to those that are present in at least half of the samples per exposure group with a differential methylation span of at least 2 adjacent windows (span of 150 bp) or 2 non-adjacent windows (span of 200 bp) within a genomic distance of 500 bp. The above filtering step was performed to minimize the sample-specific methylation variation affecting the results. In addition, using the methylation reads mapped to chromosome X and Y, the underlying methylation difference among male and female samples was distinguished and re-confirmed the sex of each mouse sample (5 male vs. 7 female). Using the mm9 Refseq annotation available from the UCSC genome browser, the gene promoters and microRNA loci within RAMs were scanned using BEDtools and in-house perl script. The complete list of RAMs and associated gene promoters and microRNA loci is available in Additional file 2: Table S3. The promoter methylation RAMs (N = 1,065, p-value & 0.05) that occur within ±1.5 kb from TSSs (mm9 Refseq) containing either low reads in at least one exposure group or at least a 5-fold change in methylation reads between any two exposure groups, were visualized using a heatmap.

### Gene set enrichment testing

The results from edgeR analysis after applying filters and removing sample-specific methylation variation resulted in 225 (Ctr vs. MG), 96 (Ctr vs. UG), and 421 (UG vs. MG) unique genes (p-value & 0.05) harboring RAMs within ±1.5 kb from TSSs. These represent the list of genes displaying altered methylation at each BPA exposure. The GO term and pathway enrichment analysis was performed using Gene Set Enricher from Comparative Toxicogenomics Database (CTD) using corrected p-value threshold of 0.05 [58,59]. A total of 60, 9, and 56 GO terms (in Ctr vs. MG, Ctr vs. UG, and UG vs. MG comparisons, respectively) were enriched, and the results were visualized using Reduce and Visualize Gene Ontology (REViGO) web application (revigo.irb.hr), which removed redundant GO terms and linked highly similar GO terms with the similarity cutoff value of 0.5 using the *Mus musculus* database [60]. Enriched GO terms and pathway analysis was also performed on the 156 known BPA-interacting genes (curated from the CTD) that are expressed in the mouse liver, obtained from the Mouse Genome Informatics Gene Expression Database using a corrected p-value of 0.01. Genome-wide region enrichment of GO terms was performed using ChIP-Enrich application using all genomic-regions (p-value & 0.05) that passed the filter for eliminating sample-specific methylation variation described above. Genome-wide region enrichment of GO terms and pathways was performed using ChIP-Enrich (http://sartorlab.ccmb.med.umich.edu/chip-enrich) package available in R software with the nearest TSS locus definition and mouse assembly (mm9) on all genomic-regions that passed the filter for eliminating sample-specific methylation variation described above.

### Quantitative methylation validation

Top candidate regions were selected based on various factors, including p-values, the number of samples with RAMs, the number of reads, and the methylation status of adjacent regions. Among the five candidate regions selected for validation, two were located within ±1.5 kb of TSSs. Genomic DNA from liver tissue from postnatal day (PND) 22 *a/a* mouse samples (N = 29), including the samples that were sequenced using M-NGS in this study, were bisulfite treated using the EpiTect bisulfite kit (Qiagen Inc., Valencia, CA) to allow for the conversion of unmethylated cytosines to uracil (read as thymine during PCR amplification), whereas the methylated cytosines remain unconverted [7]. Bisulfite converted DNA was then amplified using Bio-Rad (Model #C1000) thermal cyclers (see Additional file 2: Table S7 for primer information and PCR conditions). Amplified products were subjected to the Sequenom EpiTYPER platform (Sequenom, San Diego, CA), performed in the University of Michigan DNA Sequencing Core. For each primer set, the methylation percentage across CG sites was averaged for each sample and boxplots were used to visualize this data in Figure 5. For the primer set targeting chr18:80754900–80756100, we experienced a failed assay on 4 samples and were unable to provide boxplots with whiskers for the UG group. As the BPA exposure groups were monotonic at this locus in the M-NGS discovery stage, we pooled the UG and MG groups and used this data in Figure 5C. The differences in mean methylation levels of the samples (14 samples in Ctr, 5 in UG, and 10 in MG) in each paired group (Ctr vs. UG, Ctr vs. MG, and UG vs. MG) were tested using two-tailed t-test.

### Quantitative real-time qPCR validation

Total RNA was isolated from 10–20 mg of frozen liver from the same set of samples assayed for quantitative methylation via the RNeasy Mini kit (Qiagen, Valencia, CA) according to the manufacturer’s instructions including the optional DNase digestion step. The purity and quantity of RNA was assessed using the Nanodrop 2000 spectrophotometer (Thermo Scientific, Wilmington, DE). To produce complementary DNA for each sample, 1 μg of total RNA template was used with the iScript cDNA synthesis Kit (Bio-Rad, Hercules, CA) following the manufacturer’s protocol. The qPCR primers for *Myh7b* and *Slc22a12* were designed using GenScript Real-time PCR primer design bioinformatics tools (http://www.genscript.com). The primer sequences for RT-qPCR were as follows: *Myh7b* forward primer 5′-AGTTGGAGTTGTCCCAGGTC; *Myh7b* reverse primer 5′-TGCGCCTCAGGTTAGTACAC; *Slc22a12* forward primer 5′-CACGTGGGACCTGGTATGTA; *Slc22a12* reverse primer 5′-CCCAAACCTATCTGAGGCAT; *Gapdh* forward primer 5′-TCCATGACAACTTTGGCATTG; and *Gapdh* reverse primer 5′-CAGTCTTCTGGGTGGCAGTGA. The thermocycler settings for cDNA synthesis included incubation at 25°C for 5 min, 42°C for 60 min, and 90°C for 5 min. *Slc22a12* was not expressed in PND22 mouse liver tissue via qPCR analysis. This finding was confirmed via the Mouse Genomics Informatics database (http://www.informatics.jax.org), which reports no expression of *Slc22a12* in mouse liver. The threshold cycle (CT) was obtained for target gene *Myh7b* and reference CT was calculated for glyceraldehydes-3 phosphate dehydrogenase (*Gapdh*). Results are reported as ∆CT, which represents the difference between CT of the target gene versus the CT of the reference gene. The average ∆CT of the Ctr exposure samples were subtracted from the average ∆CT of the UG and MG exposure samples to obtain the ∆∆CT value, and fold change was calculated as 2^- ∆∆CT^.

### CpG island (CGI) annotation

The genomic coordinates for mouse CGIs (mm9) [61] were downloaded from UCSC Genome Browser. The genomic regions flanking up to 2 kb (0 – 2 kb from CGI) that do not overlap with nearby CGIs were defined as CGI shores. The genomic regions flanking up to 2 kb from CGI shores (2 - 4 kb from CGI), that do not overlap with nearby CGIs and CGI shores are defined as CGI shelves [34,62].

## Abbreviations

BPA: Bisphenol A; CGI: CpG islands; CTD: Comparative toxicogenomics database; CTR: Control (0 BPA exposure group); GD: Gestational day; M-NGS: MethylPlex-next generation sequencing; NHANES: National health and nutrition examination surveys; PND: Post-natal day; RAM: Region of altered methylation; TSS: Transcription start site; UG: μg BPA exposure group; MG: mg BPA exposure group.

## Competing interests

The authors declare that they have no competing interests.

## Authors’ contributions

JK, MAS, LSR, and DCD conceived of the study question and design. OSA, TRJ, MSN, and CF oversaw animal husbandry, performed DNA isolation, and assisted with MethylPlex library preparation. JK and MAS, with input from LSR, CF, and DCD, developed and carried out the bioinformatics pipeline to identify regions of altered methylation and test for enriched pathways. JK and DCD drafted the manuscript and created the figures with editorial guidance from MAS, LSR, and CF. All authors read and approved the final manuscript.

## Supplementary Material

Additional file 1: Figure S1 CpG Islands Are Enriched in the MethylPlex Library. MethylPlex-Next Generation Sequencing (M-NGS) reads aligned to mouse chromosome 8 are shown using the UCSC genome browser with Refseq gene and CpG Island (CGI) density. MethylPlex reads are enriched in regions containing higher numbers of genes and CGIs. **Figure S2.** MethylPlex Reads Alignment on Chromosome Y Distinguishes Males and Females. A sex-based analysis of reads aligned to the chromosome Y was performed for initial standardization of the analysis pipeline. Methylation differences are revealed between males and females in raw tag counts with minimal background in chromosome Y in females. No regions from chromosome Y were identified to harbor the hypermethylation in females after applying filters for identifying differential reads using neighboring windows and number of samples harboring the methylation in a 100 bp window. **Figure S3.** Genomic Distribution of Differentially Methylated Windows Between Male and Female Offspring. Despite limited power, sex differences are identified, and future studies with sufficient power should address sex-specific effects of exposures on the methylome. **Figure S4.** Characterization of Genome-Wide BPA Exposure Dependent Regions of Altered Methylation (RAMs). (A) Bar charts representing the genomic distribution of RAMs (p-value & 0.05) reveal both hyper- and hypomethylation across exposure. (B) Venn diagram reveals distinct RAMs across exposures. (C) Pie charts display distribution of RAMs detected in CGIs, CGI shores, and shelves. In Ctr vs. MG and UG vs. MG comparisons, approximately half of the changes occur in CGI shore (0-2kb from CGI). **Figure S5.** Pie Charts of the Genome-wide Distribution of CGIs, CGI shores, and CGI shelves in the (A) Mouse Genome and (B) Hypo and Hypermethylated Regions of Altered Methylation (RAMs). The majority of methylation changes observed in BPA-exposed mouse liver samples were located in CGI shores. Click here for file

Additional file 2: Table S1 Sample table. **Table S2.** Gender-based differentially methylation regions. **Table S3.** Candidate regions. **Table S4.** Enriched GO terms and pathways in differentially methylated regions. **Table S5.** Enriched GO terms and pathways of BPA-interacting genes. **Table S6.** ChIP-Enrich analysis results. **Table S7.** EpiTYPER primers and PCR conditions. Click here for file
