# Supplementary material for: Perinatal bisphenol A exposure promotes dose-dependent alterations of the mouse methylome
Source: BMC Genomics. 2014 Jan 17;15:30. doi: 10.1186/1471-2164-15-30 (PMC3902427; doi:10.1186/1471-2164-15-30)
Supplement: Additional file 1: Figure S1 — CpG Islands Are Enriched in the MethylPlex Library. MethylPlex-Next Generation Sequencing (M-NGS) reads aligned to mouse chromosome 8 are shown using the UCSC genome browser with Refseq gene and CpG Island (CGI) density. MethylPlex reads are enriched in regions containing higher numbers of genes and CGIs. Figure S2. MethylPlex Reads Alignment on Chromosome Y Distinguishes Males and Females. A sex-based analysis of reads aligned to the chromosome Y was performed for initial standardization of the analysis pipeline. Methylation differences are revealed between males and females in raw tag counts with minimal background in chromosome Y in females. No regions from chromosome Y were identified to harbor the hypermethylation in females after applying filters for identifying differential reads using neighboring windows and number of samples harboring the methylation in a 100 bp window. Figure S3. Genomic Distribution of Differentially Methylated Windows Between Male and Female Offspring. Despite limited power, sex differences are identified, and future studies with sufficient power should address sex-specific effects of exposures on the methylome. Figure S4. Characterization of Genome-Wide BPA Exposure Dependent Regions of Altered Methylation (RAMs). (A) Bar charts representing the genomic distribution of RAMs (p-value & 0.05) reveal both hyper- and hypomethylation across exposure. (B) Venn diagram reveals distinct RAMs across exposures. (C) Pie charts display distribution of RAMs detected in CGIs, CGI shores, and shelves. In Ctr vs. MG and UG vs. MG comparisons, approximately half of the changes occur in CGI shore (0-2kb from CGI). Figure S5. Pie Charts of the Genome-wide Distribution of CGIs, CGI shores, and CGI shelves in the (A) Mouse Genome and (B) Hypo and Hypermethylated Regions of Altered Methylation (RAMs). The majority of methylation changes observed in BPA-exposed mouse liver samples were located in CGI shores. [file 1471-2164-15-30-S1.docx]

**Supplementary Figure 1: CpG Islands Are Enriched in the MethylPlex Library.** MethylPlex-Next Generation Sequencing (M-NGS) reads aligned to chromosome 8 of mouse genome (MM9) are shown using the UCSC genome browser along with Refseq gene and CpG Island (CGI) density. MethylPlex library reads are enriched in genomic regions containing higher numbers of genes and CGIs.


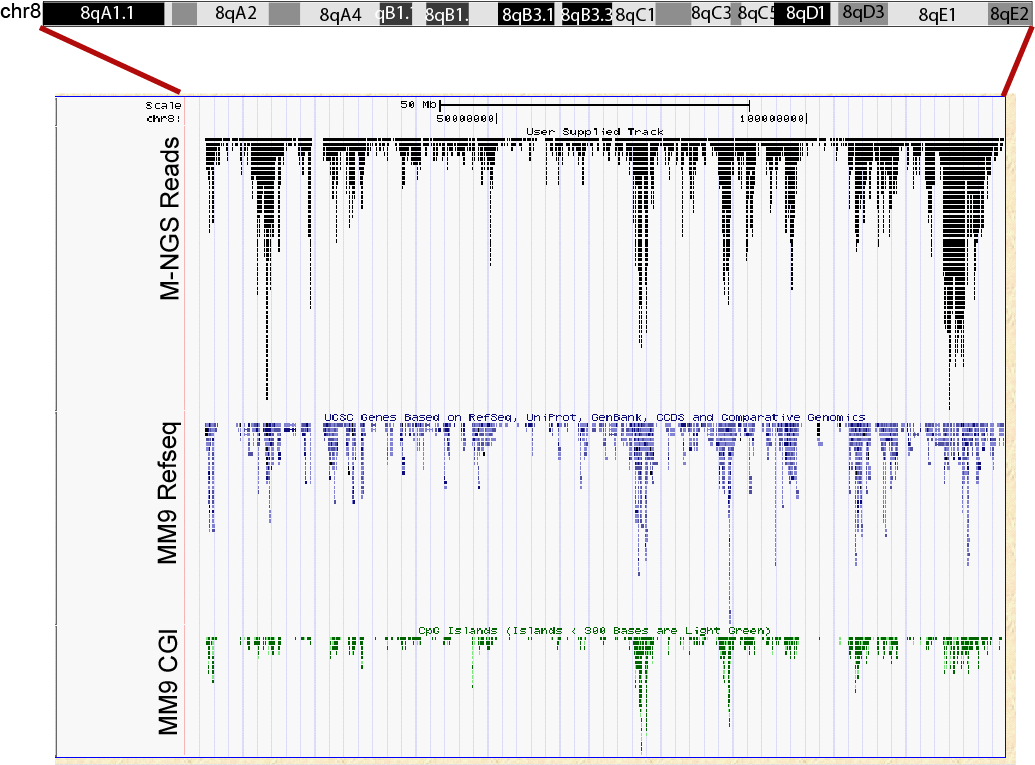


**Supplementary Figure 2: MethylPlex Reads Alignment on Chromosome Y Distinguishes Male and Female Offspring.** A sex-based analysis of MethylPlex reads aligned to the chromosome Y was performed for initial standardization of the data analysis pipeline. Methylation differences are revealed between male and female offspring in raw tag counts with minimal background noise in chromosome Y in female samples. No regions from chromosome Y were identified to harbor the hypermethylation in female samples after applying filters for identifying differential reads using neighboring windows and number of samples harboring the methylation in a given 100 bp window as described in Methods. .


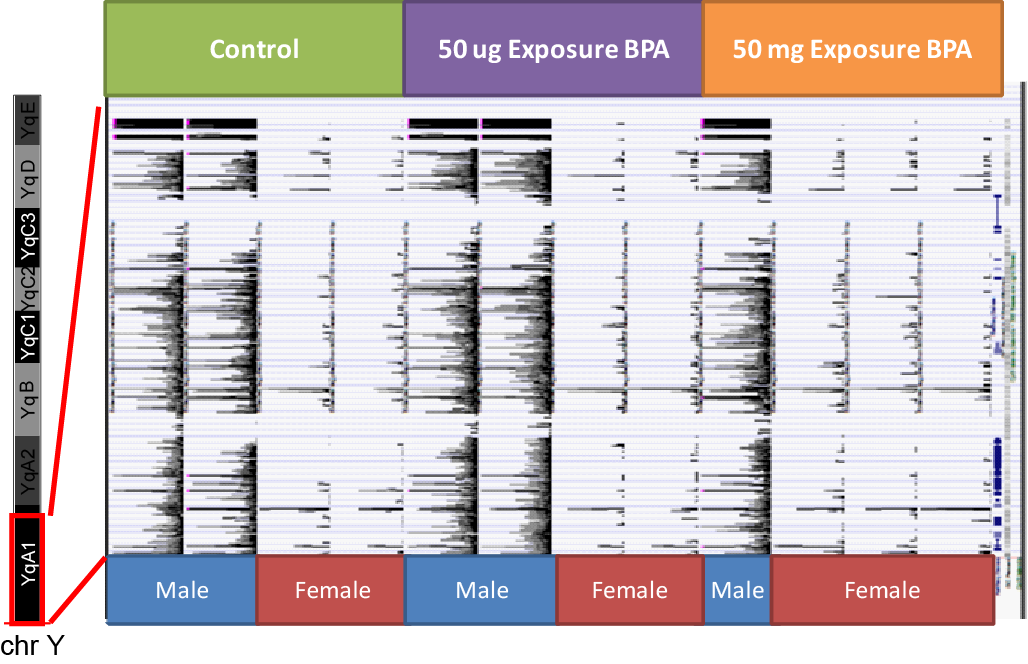


**Supplementary Figure 3: Genomic Distribution of Differentially Methylated Windows Between Male and Female Offspring.** Our experimental design was not powered to sufficiently identify sex-effects associated with BPA exposure group. Nonetheless, a number of potentially interesting sex differences are identified, and future studies with sufficient power should address sex-specific effects of exposures on the methylome.


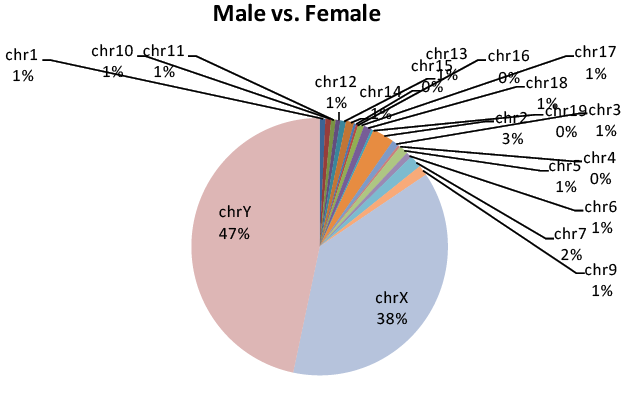


**Supplementary Figure 4: Characterization of Genome-Wide BPA Exposure Dependent Regions of Altered Methylation (RAMs).** (A) Bar charts representing the genomic distribution of RAMs (p-value < 0.05) reveal both hyper- and hypomethylation across exposure categories. (B) The Venn diagram reveals that RAMs are distinct across exposure comparisons. (C) Pie charts display the distribution of RAMs detected in CGIs, CGI shores, and shelves. In Ctr vs. MG and UG vs. MG comparisons, approximately half of the changes occur in CGI shore (0-2kb from CGI).


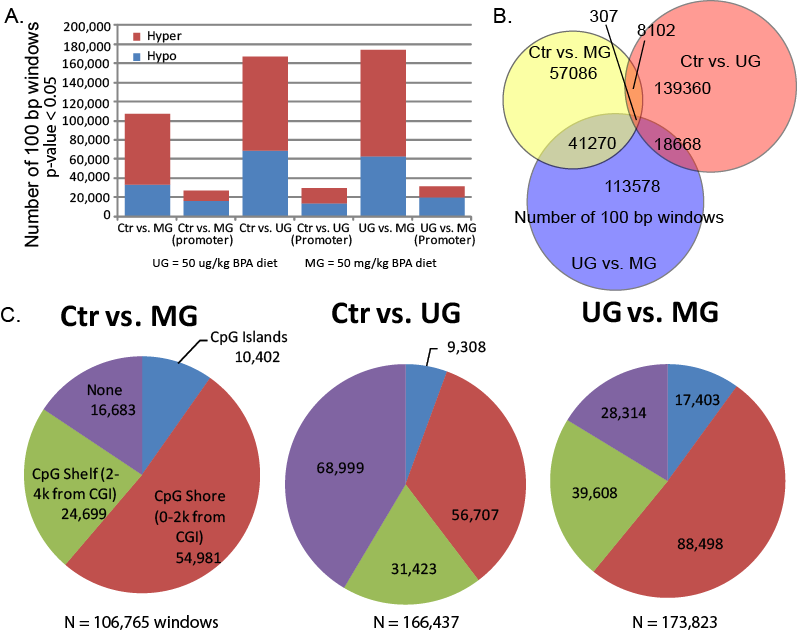


**Supplementary Figure 5:** **Pie Charts of the Genome-wide Distribution of CGIs, CGI shores, and CGI shelves in the (A) Mouse Genome (MM9) and (B) Hypo and Hypermethylated Regions of Altered Methylation (RAMs).** The majority of methylation changes observed in BPA-exposed mouse liver samples were located in CGI shores. Among hypermethylated RAMs in MG compared to Ctr group, nearly 43% of the changes resided in CGI shores, while only 12% of the hypermethylated RAMs in UG compared to Ctr group were located in CGI shores, compared to 26% in mouse genome. In CGI shelves, the hypermethylated regions in UG compared to Ctr group showed the increase in proportion at 27%, compared to 15% in mouse genome.


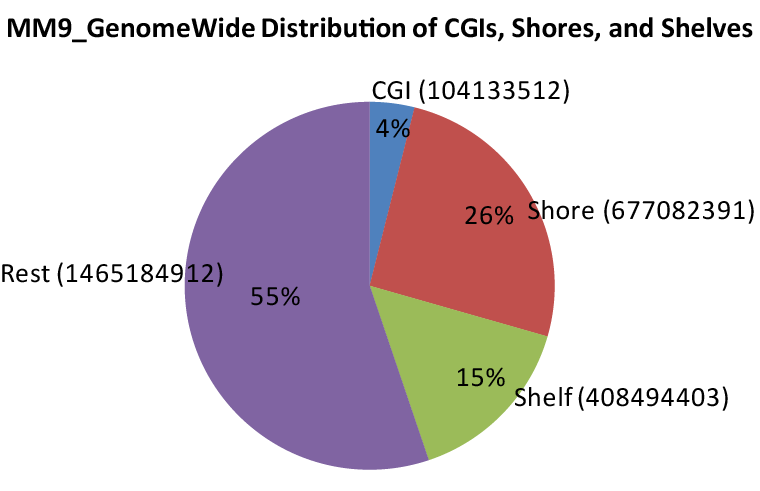
A.

B.


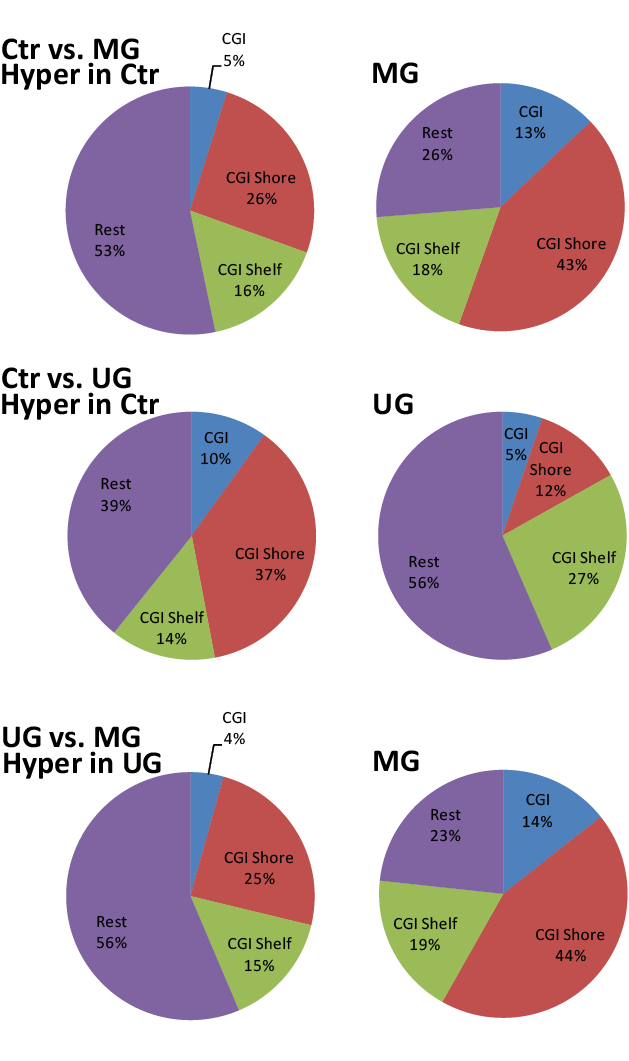


Supplementary Table 1: Sample table

Supplementary Table 2: Gender-based differentially methylation regions

Supplementary Table 3: Candidate regions

Supplementary Table 4: Enriched GO terms and pathways in differentially methylated regions

Supplementary Table 5: Enriched GO terms and pathways of BPA-interacting genes

Supplementary Table 6: ChIP-Enrich analysis results

Supplementary Table 7: EpiTYPER primers and PCR conditions
